# Supplementary material for: Comparative lipidomic profiling of African trypanosomes reveals metabolic distinctions impacting pan-species drug development
Source: PLoS Negl Trop Dis. 2026 Sep 24;20(9):e0014771. doi: 10.1371/journal.pntd.0014771 (PMC13645232; doi:10.1371/journal.pntd.0014771)
Supplement: Supplementary file 1 [file pntd.0014771.s011.pdf]

THE ROSLIN INSTITUTE
The University of Edinburgh
Easter Bush
Midlothian
EH25 9RG
Telephone: +44 (0)131 651 9100
Website : www.ed.ac.uk/roslin

14th August, 2026

Dear PLOS Neglected Tropical Diseases editorial team,

Thank you for your reviews of our manuscript entitled "Comparative lipidomic profiling of African trypanosomes reveals metabolic distinctions impacting pan-species drug development". We are pleased with the response to our work, which was generally positive. In addition, we thank the three reviewers for the useful comments they have provided that have improved the submission.

We have addressed the individual comments below and we hope that our revised manuscript is now suitable for publication in PLOS Neglected Tropical Diseases

Kind regards,

Emily Dickie

Liam Morrison

Pieter Steketee

Reviewer 1:

While data presented in this study are solid, I wonder if the same results would be obtained when the two African trypanosome species are multiplied in mouse to eliminate the differences in temperature growth requirements in in vitro culture of the parasites.

The Reviewer makes a valid point, that both T. brucei and T. congolense infect rodents possessing a core temperature of 37°C. However, in spite of our attempts (as well as those of other researchers in this field), we are currently not able to culture T. congolense at this temperature. Whilst we did not have a budget for animal experiments in this study, we agree that lipidomics analysis of parasites obtained from mouse infections would be of interest. We have added a statement alluding to this in the Discussion section of our manuscript. The statement reads as follows (lines 485-488): "Both trypanosome species infect mice, which maintain a core temperature of 37°C, and it would therefore be of interest to carry out further lipidomics analyses to ascertain whether the observations made in vitro are reflective of the in vivo situation."

Lines 161-166. the sentence is too long and difficult to understand.

Biotechnology and Biological Sciences Research Council

We have revised this statement, and split it into multiple sentences. This section now reads as follows (lines 162-165): "Lipids are often poorly detected on LC-MS metabolomics platforms using HILIC chromatography. However, peaks putatively annotated as phosphatidylethanolamines (PEs), and in addition several phosphatidylcholines (PCs) and phosphatidylinositols (PIs), were detected via LC-MS, and determined to be present only in goat serum (S1 Table)."

Line 197, delete "upon".

This has been corrected (now line 202).

Line 259-260. re-write the sentence particularly, 'with a comparative reduction'...

This sentence has been re-written. It now reads as follows (lines 266-267): "PCs, in particular short chain PCs, were consistently more abundant in T. brucei (S4 Table), compared to T. congolense cultured in both HMI-AMII and HMI-93 (Fig 4A)."

Line 367-368: delete 'these fatty acids'.

We have deleted 'these fatty acids', and replaced it with 'them' in order to maintain sentence structure (line 374)

A general comment. Some sentences in the manuscript are too long making the document difficult to understand.

We have gone through the manuscript and address long sentences. Specifically, we have re-written the following sentences:

Lines 149-152: "Serum contains sugars, such as glucose, amino acids and micronutrients such as inorganic ions and vitamins, which are supplied to considerable excess in basal media formulations. This includes IMDM, which is a main component of HMI-11 and HMI-93."

Lines 154-156: "Of the 482 metabolites detected, 285 were significantly different (BH-corrected t-test; S1 Table), of which the top 50 are shown in Fig 2A. Biologically relevant metabolites are shown in Table 1."

Lines 222-226: "Together, these data indicate that susceptibility to lipid-metabolism inhibitors is strongly shaped by the extracellular nutrient environment. They also suggest that, under HMI-AMII conditions, T. congolense retains some dependence on endogenous lipid metabolism despite remaining less sensitive than T. brucei overall."

Reviewer 2:

The most simplest part of the work, the interpretation of the proliferation curves in different sera and sera concentrations, maybe due to its low complexity, is insufficiently described. For example, the authors do not state which kind of data fitnesses they use to model the proliferation data. How did they calculate the duplication rate (did they fitd the entire proliferation curve to a linear function?, as the representation is semi-log, maybe this mask some differences, etc.). Additionally, in fig 1 the Y axis is labeled as "Cumulative cell density" while it seems to be just cell density. Please check.

We apologise that our methodology was unclear with respect to doubling time calculations. Doubling times were calculated using Microsoft Excel. Firstly, the natural log of each cell count was calculated (LN() function). We then performed a linear regression on all log-transformed cell counts from the 6-day experiment using the SLOPE() function, in order to obtain the exponential growth rate constant (k day^{-1}). To calculate doubling time, we used the formula DT = ln(2)/k × 24. As this approach assumes logarithmic growth, it does not function when cell viability

is significantly impacted (in this case, we have not calculated doubling time, as seen for samples labeled "ND" in Fig S1).

We have revised the Methods section to include a short paragraph on calculation of doubling times, which reads as follows (lines 518-522): "Doubling times were calculated using Microsoft Excel. Cell densities were log-transformed using the LN() function and a linear regression was performed using the SLOPE() function to calculate the exponential growth rate constant (k day^{-1}). Doubling time was then calculated using the formula DT = ln(2)/k × 24. Where cell viability was significantly impacted, doubling time was impossible to determine. These samples are labelled as "ND"."

With respect to the "cumulative cell density" Y axis labels in Fig 1, this is indeed similar to cell density. However, over 7 days of incubation (day 0 to day 6), both trypanosome species need to be passaged at least once or twice to avoid the culture exhausting. We take the dilution factors (culture volume / seeding volume added to the culture) into account and multiply subsequent cell counts by this value to obtain the cumulative cell density. This ensures that a) parasites remain in log phase during the experiment and b) graphed data is easier to view on a log-axis.

We have further updated the Methods section to include a sentence on calculating the cumulative cell density for growth curve figures. This sentence reads as follows (lines 515-517): "Cumulative cell density was calculated by taking dilution factors (culture volume/cell seeding volume) in account. Subsequent cell counts were multiplied by these dilution factors to generate cumulative cell density values that were plotted.".

In Fig. 2B, the authors used FBS supplemented with lipid-loaded BSA. They should justify or discuss the absence of a control with BSA not loaded with lipids.

We agree with the Reviewer that a BSA-only control would have benefitted the study. However, we note that the lipid-loaded BSA used in this study is a proprietary product developed commercially (albuMAX II; Gibco), which means that it is impossible to obtain the chemically analogous BSA without coupled lipids. This is critical, as BSA batches are known to vary in their purity and lipid content. Our groups are currently attempting to develop culture systems that do not require the use of proprietary products. If successful, BSA-only controls will be included in these experiments. However, on this occasion, it was impossible to include this kind of control as it would likely introduce confounding variables due to BSA bath variation.

We have edited the main text to reflect this (lines 192-194): "Notably, it was not possible to compare growth in AMII to BSA-only controls, as this supplement is pre-coupled in a proprietary manner, and BSA is known to vary depending on batch and origin."

A suggestion: all along the manuscript, the authors use extensively the term "growth" and derivatives... maybe it is more appropriate to use "proliferation".

We agree with the Reviewer's suggestion. We have gone through the manuscript and changed instances of "growth" to "proliferation"

The Roslin Institute, a world class research centre, receives strategic investment funding from BBSRC
and it is part of the University of Edinburgh's Royal (Dick) School of Veterinary Studies.
The University of Edinburgh is a charitable body, registered in Scotland, with registration number SC005336

UKRI Biotechnology and Biological Sciences Research Council

Reviewer 3:

It may have been helpful if the comparative metabolomic analysis had been extended to horse and calf, as Tc seems to grow close to GS20% in these sera. These additional data could produce a pattern that might help reinforce the findings of the study.

We agree with the Reviewer that further metabolomics analyses of other serum types would strengthen the work. Unfortunately, these experiments are costly, and therefore, our research budget could only cover the goat serum vs FBS comparison shown in our manuscript. Comparative analysis of more sera from diverse sources is certainly something we would be interested to carry out in the future, and something that would be useful to the field.

L 145; clarify what is meant by long-term culture in FBS, as growth seems reasonable over 6 days in 10% FBS

We apologies for the lack of clarity. We would define long term culture as cells that are cultured up to 20 passages, which is typically the maximum length of time we would culture both parasite species to avoid significant genetic mutations associated with medium adaptation.

We have clarified this statement in the manuscript. The sentence now reads as follows (lines 145-146): "However, long-term culture in FBS (up to 20 passages) was not successful."

L151 it would be useful if composition of HMI-11 v -93 were given in an appendix.

This is a good suggestion from the Reviewer. We have now added an extra supplementary table (S2 Table) showing the composition of both HMI-11 and HMI-93. We have additionally included HMI-AMII in this table. Please note that all three media are IMDM-based (as stated in the new table), so the majority of medium components do not vary between the media.

We have added text to the Methods section highlighting the new supplementary table. This text reads as follows (line 511): "Medium formulations are provided in S2 Table."

L166, S1 table, this table needs a legend and some explanation of how the Log2Fold values are calculated. For example for cholate this value is 19.78, or 900,271 fold (is this the case?)

Apologies for the confusion. To calculate Log_2 fold changes in metabolites between goat serum and FBS, we first log-transformed (Log_2) the raw peak intensity counts for each metabolite. However, many metabolites were only detected in one of the two sample groups, with the undetected value equalling 0. As Log_2 of zero is a negative infinity, we elected to calculate Log_2 of each peak intensity + 1 (Log2 of 1 is zero). We then calculated Log_2 fold change (Log_2FC) by subtracting Log2_FBS from Log2_GS. In the case of metabolites that were only detected in one sample group (including cholate – only detected in goat serum) this can lead to artificially high Log_2FC values. For this reason, we included the raw intensity and log-transformed intensities in the supplementary table, so that the reader is able to compare Log_2FC values to the raw peak intensities for metabolites of interest.

We have now added a legend explaining the calculations carried out to generate the Log_2FC values. This legend reads as follows (lines 764-774): "A total of 482 metabolites were putatively identified in one or both sera. Column A: putative identities of the metabolites; columns B-E: raw peak intensities for four technical replicates of goat serum; columns F-I: raw peak intensities for four technical replicates of FBS; columns J-Q: Log_2-transformed peak intensities for goat serum

(J-M) and FBS (N-Q). The transformation was carried out on peak intensities + 1 to ensure that undetected metabolites (peak intensity = 0) returned a value of 0; column R: Log_2 fold change between goat serum and FBS, calculated by subtracting the mean Log_2 FBS from the mean Log_2 goat serum values. Column S: t-test result for each Log_2FC, calculated using Metaboanalyst; column T: FDR-adjusted P value (t-test). Abbreviations: GS: goat serum; FBS: foetal bovine serum; Log_2FC: Log_2 fold change; FDR: false discovery rate."

L161 -166, Clarify: is the relatively poor detection of lipids using HILIC a limitation and if so does this affect the interpretation that the differences between growth in GS v FBS are due to lipids?

We agree that HILIC systems, coupled to our typical metabolite extraction methods (chloroform:methanol:water extraction) are optimised for the extraction of polar metabolites, and are less effective for the extraction of hydrophobic lipid species. This approach was therefore not ideal for analysing lipid differences between the two sera as the identifications are not as accurate compared to approaches involving lipidomics extraction methods (akin to those used for the T. congolense vs. T. brucei lipidomics comparisons).

However, we believe this experiment was still very useful as, whilst it highlighted some differences in polar metabolites between the sera, the majority of significant differences were putative lipid species that could not be accurately identified. This led us to the hypothesis that adding extra exogenous lipid sources would enable T. congolense growth in FBS-supplemented medium, as proven in Fig 2B.

L191 and Fig. 2B growth rates (by eye) in Fig 2B seems to be fastest for FBS (10%) + aMMII, yet DT is 9.5 h v 9.1 for GS20%. It is certainly clearer there is a difference in the opposite way around day 3 to 6. Also be consistent with presentation, AMMII or aMMII not both.

The Reviewer has highlighted an error in our figure that we did not notice prior to our first submission. We sincerely apologise for this. We have revised the T. congolense graph (Fig 2B) with the correct data – the same data from which the doubling times were calculated. The previous graph showed T. brucei data, which is erroneous. We note that this revision does not affect the overall conclusions of this study. We thank the Reviewer for highlighting this inconsistency.

The doubling times for these analyses were calculated over the entire growth curve (a total of 7 days, including seeding at day 0; please refer to our answer to a similar question posed by Reviewer 2 for more detail), as this encompasses biological variation to a greater degree.

We have corrected the text error regarding "aMII" (Fig 2B and 2C), which now reads "HMI-AMII" to ensure consistency with the rest of the manuscript. We thank the Reviewer for raising this issue in addition to the other issue raised with regards to Fig 2 (see below).

L201 -204 and Fig 2 D & E. Clarify what is meant by each species standard media. As written, it is not clear if the drug sensitivity assays were performed in serum free media. Does the media as presented in Fig D and E serum free, probably but it is not clear? If so what is the effect on cell survival for 48 h under serum free conditions.

We apologise for any confusion with regards to the drug sensitivity assays. By each species' standard medium we were referring to HMI-93 for T. congolense and HMI-11 for T. brucei – these are the media that these parasites are typically cultured in. The drug sensitivity assays were carried out in these media in addition to HMI-AMII (containing 10% FBS). This was done to confirm whether the new medium formulation led to any phenotypic changes. We have revised the Results

and Methods sections to emphasise that these drug sensitivity assays were carried out in HMI-11 (10% FBS), HMI-93 (20% goat serum) and HMI-AMII (10% FBS). Specifically:

Lines 202-208: "To determine whether the altered medium conditions impacted T. brucei and T. congolense lipid metabolism, parasite sensitivity to the metabolic inhibitors acetyl-CoA synthetase (ACS-In) and Orlistat were carried out in HMI-93 (T. congolense), HMI-11 (T. brucei) and HMI-AMII (both species). Consistent with previous findings [10], T. congolense remained less sensitive to both inhibitors in HMI-AMII (Fig 2D & 2E). However, comparisons between HMI-AMII and species-specific culture medium (HMI-93 and HMI-11 for T. congolense and T. brucei, respectively) revealed clear medium-dependent shifts with regards to inhibitor sensitivity."

Lines 533-537: "Parasites were cultured in their standard media (HMI-11 and HMI-93 for T. brucei and T. congolense, respectively) or HMI-AMII for at least two passages prior to harvesting at log-phase of growth, and distributed across the wells (100 µL/well) at final concentrations of 2 × 10^4 and 2.5 × 10^5 cells/mL for T. brucei and T. congolense, respectively, in their corresponding culture media."

In addition to lipids, serum is an important source of vitamins, co-factors, trace minerals and proteins that enable cell viability under in vitro conditions. Whilst we have not specifically tested the proliferation of our trypanosome lines in serum free medium, it is well established that long-term (up to 20 passages) culture in serum free conditions is not possible for both T. congolense and T. brucei. We would hypothesise that serum free conditions for 48 h would cause a significant amount of stress on the cells, which would negatively impact drug sensitivity interpretations.

Does Tc HMI-93in Fig2D contain GS and similarly for HMI-11 for Tb. The legend to Fig should properly be TcHMI-93-AMII (93 is missing, similarly for MII-11..is this the case or is HMI-AMII something different , see also line 239).

We apologise for the lack of clarity in this figure and associated text, this was an oversight on our part. In these experiments, we found that 10% FBS and 2.5% AMII were sufficient for the culture of both species, using a medium based on HMI-11, to ensure consistency between the species. HMI-11 only differs slightly from HMI-93 – the concentrations of hypoxanthine, L-cysteine, 2-mercaptoethanol and serum source/concentration. These differences can be seen in the new S2 Table.

Figs 2B, 2C and 2D therefore employ a universal medium, which we have termed HMI-AMII. To ensure consistency, we have updated the legends for Figs 2B and 2C. We apologise that this was not clear in our first submission.

To further clarify this, we have substantially revised a portion of the Results section (lines 186-201): "To probe this further, viability of both species was assessed when grown in medium supplemented with FBS and a commercially available lipid-loaded BSA supplement (albuMAX II, AMII; 2.5% v/v; Fig 2B & 2C). To ensure consistency, a single HMI-11-based medium – termed HMI-AMII – was used for both species (note that standard HMI-11 and HMI-93 differ only in serum requirements and in the concentrations of L-cysteine, hypoxanthine and 2-mercaptoethanol; S6 Table). Control cultures were maintained in their standard species-specific media (HMI-93 for T. congolense, Fig 2B; HMI-11 for T. brucei, Fig 2C). Notably, it was not possible to compare growth in AMII to BSA-only controls, as this supplement is pre-coupled in a proprietary manner, and BSA is known to vary depending on batch and origin.

In HMI-AMII supplemented with 20% and 10% FBS, T. congolense grew at rates comparable to that of the goat serum control (mean doubling time of 10.03 hr, 9.5

hr and 9.1 hr for 20% FBS, 10% FBS and 20% GS, respectively), suggesting the addition of the BSA supplement was key to proliferation of this species in FBS-supplemented medium in vitro (Fig 2B). Cell viability of T. brucei was not impacted by lipid supplementation, indicating that HMI-AMII medium supplemented with 10% FBS and 2.5% AMII (HMI-AMII) was sufficient for the culture of both trypanosome species (Fig 2C)."

We have revised line 239 to be more consistent (now line 244-246): "In order to compare differences in lipid composition further, untargeted LC-MS lipidomics analysis was performed on T. brucei (cultured in HMI-AMII) and T. congolense BSFs (cultured in HMI-93 with 20% goat serum as well as HMI-AMII)…."

It seems counter intuitive that Tc becomes more sensitive to inhibitors of acetyl Co A synthase and lipases when the media was supplemented with albuMAXII. This is especially true for Orilstat. Why would they become more sensitive to lipase inhibition(decreased hydrolysis of triglycerides) when the media is supplemented with additional lipids , fatty acids and triglycerides?

We agree that these results seem counterintuitive, and can only hypothesise as to the underlying reasons. Firstly, it is important to note that when cultured in HMI-93, T. congolense has a 20% supplement of a lipid-rich serum (goat serum). When switched to HMI-AMII, there is 10% FBS and 2.5% lipid-rich BSA. Therefore, overall lipid abundance is likely lower, resulting in a requirement for lipid synthesis. Furthermore, it could be that the lipid species preferentially scavenged by T. congolense are present in goat serum, but not to the same degree in FBS or albuMAX II, which would again lead to an increased reliance on de novo synthesis (unfortunately, albuMAX II is a proprietary product, and we therefore do not know the exact lipid composition of this supplement).

Alternatively, orlistat is a highly lipophilic drug. With a reduced lipid abundance in the culture (under the 10% FBS; 2.5% albuMAX II conditions), there may be more free drug targeting the parasite, leading to the appearance of increased drug sensitivity for T. congolense. Whilst these theories are all plausible, we feel it is outside the remit of this study to determine the specific underlying reason.

L361-362 and Table 2. Clarify the media conditions for data in Table 2. Is this HMI-11 /HMI-93 with 10% of the relevant serum FBS/GS?

We apologise for the lack of clarity in this table legend. The parasites were harvested from the same medium formulations as the lipidomics analysis presented earlier in the manuscript. Thus, T. brucei was cultured in HMI-AMII and T. congolense was cultured in either HMI-AMII or HMI-93. We have revised the table's column headings to make this clearer.

We have also expanded the legend in order to clarify this. The table legend now reads as follows (lines 384-387): "Table 2: Comparative GC-MS fatty acid analysis of T. brucei and T. congolense. The culture medium used for each sample group is indicated in the column title (HMI-AMII: 10% FBS, 2.5% AMII; HMI-93: 20% goat serum). Abundance of each FA is shown as a percentage of total detected FA."

Also, the major difference between the two species appears to be C22:5, perhaps this should be noted

We agree that C22:5 is a major difference. However, there are also other fatty acids that show a similar species-specific difference (regardless of culturing conditions for T. congolense): C16:0, C18:0, C18:3 and C20:3. Nevertheless, we have added in a statement highlighting C22:5 and the aforementioned fatty acids as showing a species-specific difference. The statement reads as follows (lines

The Roslin Institute, a world class research centre, receives strategic investment funding from BBSRC
and it is part of the University of Edinburgh's Royal (Dick) School of Veterinary Studies.
The University of Edinburgh is a charitable body, registered in Scotland, with registration number SC005336
Biotechnology and Biological Sciences Research Council

380-381): "The most significant species-specific differences were observed for C16:0, C18:0, C18:3, C20:3 and C22:5 (Table 2)."

Overall, the data are sound and the conclusions are reasonable. Perhaps the insights are not of the highest significance, but they are worthwhile and add to our understanding of a neglected area of study in these parasites. They also align with RNAseq data and One element that is not really discussed is how lipid up take and acquisition might affect the lipid profile. Perhaps too the difference in culture temperature reflects these differences for example via fluidity of the surface membrane. This point is mentioned briefly at the end of the discussion but could developed more.

Thanks to the Reviewer for this suggestion. However, we already felt the Discussion section was long, and did not want to expand on too many hypotheticals. We believe we have adequately addressed the fluidity of the surface membrane in lines 468-479 and the difference in culture temperature in lines 480-488.
