# Supplementary material for: Comparative lipidomic profiling of African trypanosomes reveals metabolic distinctions impacting pan-species drug development
Source: PLoS Negl Trop Dis. 2026 Sep 24;20(9):e0014771. doi: 10.1371/journal.pntd.0014771 (PMC13645232; doi:10.1371/journal.pntd.0014771)
Supplement: Peer review history

# Peer review: Decision Letter 0

**DOI:** 10.1371/journal.pntd.0014771.r001

**Authors:** Brian L Weiss, Susan Madison-Antenucci

11 Aug 2026

Response to Reviewers Revised Manuscript with Track Changes Manuscript

Shaden Kamhawi

co-Editor-in-Chief

PLOS Neglected Tropical Diseases

orcid.org/0000-0003-4304-636XX

Paul Brindley

co-Editor-in-Chief

PLOS Neglected Tropical Diseases

orcid.org/0000-0003-1765-0002

**Journal Requirements:**

1) Please amend your detailed Financial Disclosure statement. This is published with the article. It must therefore be completed in full sentences and contain the exact wording you wish to be published.

i) State what role the funders took in the study. If the funders had no role in your study, please state: "The funders had no role in study design, data collection and analysis, decision to publish, or preparation of the manuscript."

ii) If any authors received a salary from any of your funders, please state which authors and which funders..

If you did not receive any funding for this study, please simply state: u201cThe authors received no specific funding for this work.u201d

2) Your current Financial Disclosure states, " E.D. was supported by a Scottish Metabolomics Network Training grant, a Wellcome ISSF feasibility grant, and funding from the Scottish Universities Life Sciences Alliance. P.S. is supported by a BBSRC Discovery Fellowship (BB/X009807/7). This study was partially supported by a BBSRC grant awarded to L.M. and M.B. (BB/S00243X/1, BB/S001034/1). The Roslin Institute is supported through core funding from the BBSRC (BS/E/D/20002173; BBS/E/RL/230002C).".However, your funding information on the submission form indicates "Order of grants" mismatches. Please indicate by return email the full and correct funding information for your study and confirm the order in which funding contributions should appear. Please be sure to indicate whether the funders played any role in the study design, data collection and analysis, decision to publish, or preparation of the manuscript.

3) We have amended your Competing Interest statement to comply with journal style. We kindly ask that you double check the statement and let us know if anything is incorrect.

**Reviewers' comments:**

**Key Review Criteria Required for Acceptance?**

As you describe the new analyses required for acceptance, please consider the following:

**Methods**

-Are the objectives of the study clearly articulated with a clear testable hypothesis stated?

-Is the study design appropriate to address the stated objectives?

-Is the population clearly described and appropriate for the hypothesis being tested?

-Is the sample size sufficient to ensure adequate power to address the hypothesis being tested?

-Were correct statistical analysis used to support conclusions?

-Are there concerns about ethical or regulatory requirements being met?

Reviewer #1: -Are the objectives of the study clearly articulated with a clear testable hypothesis stated? Yes

-Is the study design appropriate to address the stated objectives? Yes

-Is the population clearly described and appropriate for the hypothesis being tested? N/A

-Is the sample size sufficient to ensure adequate power to address the hypothesis being tested? Yes

-Were correct statistical analysis used to support conclusions? Yes

-Are there concerns about ethical or regulatory requirements being met? N/A

Reviewer #2: This study starts based on the observatin that Trypanosoma brucei can be cultured in 10% FCS, while Trypanosoma congolense must be cultured in goat serum 20%, indicating that the metabolites supplemented to the base medium by the sera are critical for their respective proliferation. The topic is extremely reelvant in view of the development of trypanocidal drugs able to tackle human and veterinary Trypanosomes. The experimental approach is original and very approppriate, very well aligned with the goals of the study. The results are clearly described and I could not detect overstatements. The discussion is rich in content and frames the work very adequately in the literature. As there were not used animals or human subjects in this study, there are no concerns about ethical or regulatory requirements.

Reviewer #3: No issues. The methodology is appropriate and well described.

**********

**Results**

-Does the analysis presented match the analysis plan?

-Are the results clearly and completely presented?

-Are the figures (Tables, Images) of sufficient quality for clarity?

Reviewer #1: Yes

Reviewer #2: As mentioned above, yes, yes and yes.

Reviewer #3: The results are clearly presented and easy to follow. There are some minor clarifications required and some formatting issues that need to be addressed and are detailed in the relevant section below.

**********

**Conclusions**

-Are the conclusions supported by the data presented?

-Are the limitations of analysis clearly described?

-Do the authors discuss how these data can be helpful to advance our understanding of the topic under study?

-Is public health relevance addressed?

Reviewer #1: -Are the conclusions supported by the data presented? Yes

-Are the limitations of analysis clearly described? Yes

-Do the authors discuss how these data can be helpful to advance our understanding of the topic under study? Yes

-Is public health relevance addressed? Yes

Reviewer #2: As mentioned above, I could not detect overstatements, so the conclusions are supported by the experimental data and the context contributed by the cited references. In the discusion, some limitations of the study are pointed out. As mentioned above, the discussion is very rich, and it states clearly the contribution of the work to the advancement of the understanding of the topic under study.

Reviewer #3: It may have been helpful if the comparative metabolomic analysis had been extended to horse and calf, as Tc seems to grow close to GS20% in these sera. These additional data could produce a pattern that might help reinforce the findings of the study.

Overall the data support the conclusions and provide further insight to previously published RNAseq data , ref 10.

**********

**Editorial and Data Presentation Modifications?**

Use this section for editorial suggestions as well as relatively minor modifications of existing data that would enhance clarity. If the only modifications needed are minor and/or editorial, you may wish to recommend “Minor Revision” or “Accept”.

Reviewer #1: Minor Revision

Reviewer #2: Some minor modifications and suggestions:

1. The most simplest part of the work, the interpretation of the proliferation curves in different sera and sera concentrations, maybe due to its low complexity, is insufficiently described. For example, the authors do not state which kind of data fitnesses they use to model the proliferation data. How did they calculate the duplication rate (did they fitd the entire proliferation curve to a linear function?, as the representation is semi-log, maybe this mask some differences, etc.). Additionally, in fig 1 the Y axis is labeled as "Cumulative cell density" while it seems to be just cell density. Please check.

2. In Fig. 2B, the authors used FBS supplemented with lipid-loaded BSA. They should justify or discuss the absence of a control with BSA not loaded with lipids.

A suggestion: all along the manuscript, the authors use extensively the term "growth" and derivatives... maybe it is more approppriate to use "proliferation".

Reviewer #3: L 145; clarify what is meant by long-term culture in FBS, as growth seems reasonable over 6 days in 10% FBS

L151 it would be useful if composition of HMI-11 v -93 were given in an appendix,

L166, S1 table, this table needs a legend and some explanation of how the Log2Fold values are calculated. For example for cholate this value is 19.78, or 900,271 fold (is this the case?)

L161 -166, Clarify : is the relatively poor detection of lipids using HILIC a limitation and if so does this affect the interpretation that the differences between growth in GS v FBS are due to lipids?

L191 and Fig. 2B growth rates (by eye) in Fig 2B seems to be fastest for FBS (10%) + aMMII, yet DT is 9.5 h v 9.1 for GS20%. It is certainly clearer there is a difference in the opposite way around day 3 to 6. Also be consistent with presentation, AMMII or aMMII not both.

L201 -204 and Fig 2 D & E. Clarify what is meant by each species standard media. As written, it is not clear if the drug sensitivity assays were performed in serum free media. Does the media as presented in Fig D and E serum free, probably but it is not clear? If so what is the effect on cell survival for 48 h under serum free conditions.

Does Tc HMI-93in Fig2D contain GS and similarly for HMI-11 for Tb. The legend to Fig should properly be TcHMI-93-AMII (93 is missing, similarly for MII-11..is this the case or is HMI-AMII something different , see also line 239).

It seems counter intuitive that Tc becomes more sensitive to inhibitors of acetyl Co A synthase and lipases when the media was supplemented with albuMAXII. This is especially true for Orilstat. Why would they become more sensitive to lipase inhibition( decreased hydrolysis of triglycerides) when the media is supplemented with additional lipids , fatty acids and triglycerides?

L361-362 and Table 2. Clarify the media conditions for data in Table 2. Is this HMI-11 /HMI-93 with 10% of the relevant serum FBS/GS?

Also the major difference between the two species appears to be C22:5, perhaps this should be noted

**********

**Summary and General Comments**

Use this section to provide overall comments, discuss strengths/weaknesses of the study, novelty, significance, general execution and scholarship. You may also include additional comments for the author, including concerns about dual publication, research ethics, or publication ethics. If requesting major revision, please articulate the new experiments that are needed.

Reviewer #1: In this manuscript, Dickie et al report a study on comparative analysis on lipidomic profiles of two different African trypanosome species. The study reports on a novel in vitro culture medium that can be used to culture the two different African trypanosome parasite species previously not possible. This work is valuable, especially when you consider the differences in the in vitro culture requirements for African trypanosomes, where data are assessed after in vitro proliferation of T. brucei and T. congolense in the same medium. They find some striking difference in lipid preference between T. brucei and T. congolense that forms a foundation for discovery and design of novel drugs against both African trypanosome parasites. The manuscript is very well-written and provides a large panel of clear, complementary and solid data underlying the author’s conclusions that are discussed in a fairly manner. I only have few comments and suggestions below.

Comments and suggestions

• While data presented in this study are solid, I wonder if the same results would be obtained when the two African trypanosome species are multiplied in mouse to eliminate the differences in temperature growth requirements in in vitro culture of the parasites.

• Lines 161-166. the sentence is too long and difficult to understand.

• Line 197, delete “upon”.

• Line 259-260. re-write the sentence particularly, ‘with a comparative reduction’...

• Line 367-368: delete ‘these fatty acids’.

• A general comment. Some sentences in the manuscript are too long making the document difficult to understand.

Reviewer #2: Overall is a relevant work on a relevant topic. It has been very well conducted, with impactful results. Additionally, it brings to the trypanosome community several useful OMICs datasets.

Reviewer #3: This study investigates the growth of two species of African trypanosome in different sera and why growth of T. brucei is favoured in FBS while T. congolense is favoured in goat serum. The justification is that these serum preferences point to metabolic distinctions between the two pathogens that maybe important for the development of pan species therapies for human and animal trypanosomiasis. The focus of the study is the lipidomic profile of the parasites grown in the media with different serum supplementation. The lipid profile of the various sera is also investigated.

The major findings are

(i) The most prominent difference between the two species was in relative abundance of ether glycerophospholipids, which are far less abundant in Tc compared to Tb.

(ii) The Tb lipidome has a preponderance of shorted chain saturated lipids whereas the Tc lipidome revealed a preference for longer chain polyunsaturated fatty acids

(iii) Tc cultured in GS exhibited a higher abundance of several LPCs compared to either species in FBS. So intrinsic differences remain even when cultured in the same media.

Taken together the authors conclude that the data indicate significant differences in the pathways for lipid synthesis and remodelling in these pathogens that is linked to evolutionary divergence between the two species.

Overall, the data are sound and the conclusions are reasonable. Perhaps the insights are not of the highest significance, but they are worthwhile and add to our understanding of a neglected area of study in these parasites. They also align with RNAseq data and

One element that is not really discussed is how lipid up take and acquisition might affect the lipid profile. Perhaps too the difference in culture temperature reflects these differences for example via fluidity of the surface membrane. This point is mentioned briefly at the end of the discussion but could developed more.

**********

PLOS authors have the option to publish the peer review history of their article (what does this mean?). If published, this will include your full peer review and any attached files.

If you choose “no”, your identity will remain anonymous but your review may still be made public.

**Do you want your identity to be public for this peer review?**  For information about this choice, including consent withdrawal, please see our Privacy Policy.

Reviewer #1: No

Reviewer #2: No

Reviewer #3: No

**Figure resubmission:**

**Reproducibility:**

To enhance the reproducibility of your results, we recommend that authors of applicable studies deposit laboratory protocols in protocols.io, where a protocol can be assigned its own identifier (DOI) such that it can be cited independently in the future. Additionally, PLOS ONE offers an option to publish peer-reviewed clinical study protocols. Read more information on sharing protocols at https://plos.org/protocols?utm_medium=editorial-email&utm_source=authorletters&utm_campaign=protocols

# Author comment: Author response to Decision Letter 1

**DOI:** 10.1371/journal.pntd.0014771.r002

17 Aug 2026

Attachment Submitted filename: TbTc_lipidomics_reviewer_responses.pdf

# Editor report: Decision Letter 1

**DOI:** 10.1371/journal.pntd.0014771.r003

**Authors:** Brian L Weiss, Susan Madison-Antenucci

10 Sep 2026

Dear Steketee,

We are pleased to inform you that your manuscript 'Comparative lipidomic profiling of African trypanosomes reveals metabolic distinctions impacting pan-species drug development' has been provisionally accepted for publication in PLOS Neglected Tropical Diseases.

Before your manuscript can be formally accepted you will need to complete some formatting changes, which you will receive in a follow up email. A member of our team will be in touch with a set of requests.

Please note that your manuscript will not be scheduled for publication until you have made the required changes, so a swift response is appreciated.

IMPORTANT: The editorial review process is now complete. PLOS will only permit corrections to spelling, formatting or significant scientific errors from this point onwards. Requests for major changes, or any which affect the scientific understanding of your work, will cause delays to the publication date of your manuscript.

Should you, your institution's press office or the journal office choose to press release your paper, you will automatically be opted out of early publication. We ask that you notify us now if you or your institution is planning to press release the article. All press must be co-ordinated with PLOS.

Thank you again for supporting Open Access publishing; we are looking forward to publishing your work in PLOS Neglected Tropical Diseases.

Best regards,

Brian L. Weiss

Academic Editor

PLOS Neglected Tropical Diseases

Susan Madison-Antenucci

Section Editor

PLOS Neglected Tropical Diseases

Shaden Kamhawi

co-Editor-in-Chief

PLOS Neglected Tropical Diseases

orcid.org/0000-0003-4304-636XX

Paul Brindley

co-Editor-in-Chief

PLOS Neglected Tropical Diseases

orcid.org/0000-0003-1765-0002

***********************************************************

# Editor report: Acceptance letter

**DOI:** 10.1371/journal.pntd.0014771.r004

**Authors:** Brian L Weiss, Susan Madison-Antenucci

Dear Steketee,

We are delighted to inform you that your manuscript, "Comparative lipidomic profiling of African trypanosomes reveals metabolic distinctions impacting pan-species drug development," has been formally accepted for publication in PLOS Neglected Tropical Diseases.

We have now passed your article onto the PLOS Production Department who will complete the rest of the publication process. All authors will receive a confirmation email upon publication.

The corresponding author will soon be receiving a typeset proof for review, to ensure errors have not been introduced during production. Please review the PDF proof of your manuscript carefully, as this is the last chance to correct any scientific or type-setting errors. Please note that major changes, or those which affect the scientific understanding of the work, will likely cause delays to the publication date of your manuscript. Note: Proofs for Front Matter articles (Editorial, Viewpoint, Symposium, Review, etc...) are generated on a different schedule and may not be made available as quickly.

Soon after your final files are uploaded, the early version of your manuscript will be published online unless you opted out of this process. The date of the early version will be your article's publication date. The final article will be published to the same URL, and all versions of the paper will be accessible to readers.

For Research Articles, you will receive an invoice from PLOS for your publication fee after your manuscript has reached the completed accept phase. If you receive an email requesting payment before acceptance or for any other service, this may be a phishing scheme. Learn how to identify phishing emails and protect your accounts at https://explore.plos.org/phishing.

Thank you again for supporting open-access publishing; we are looking forward to publishing your work in PLOS Neglected Tropical Diseases.

Best regards,

Shaden Kamhawi

co-Editor-in-Chief

PLOS Neglected Tropical Diseases

Paul Brindley

co-Editor-in-Chief

PLOS Neglected Tropical Diseases
